# Inferior stabilization of cementless compared with cemented dual-mobility cups in elderly osteoarthrosis patients: a randomized controlled radiostereometry study on 60 patients with 2 years’ follow-up

**DOI:** 10.1080/17453674.2020.1720978

**Published:** 2020-02-06

**Authors:** Steffan Tabori-Jensen, Sebastian Breddam Mosegaard, Torben B Hansen, Maiken Stilling

**Affiliations:** a University Clinic for Hand, Hip and Knee Surgery, Regional Hospital West Jutland, Holstebro;;; b Department of Clinical Medicine, Aarhus University, Aarhus, Denmark

## Abstract

Background and purpose — Elderly patients may benefit from a dislocation low-risk dual-mobility (DM) articulation in total hip arthroplasty, but the best cup fixation method is unknown. We compared cup migration for cemented and cementless DM cups using radiostereometry.

Patients and methods — In a patient-blinded randomized trial, 60 patients (33 female) with osteoarthritis were allocated to cemented (n = 30) or cementless (n = 30) Avantage DM cup fixation. Criteria were age above 70 years, and T-score above –4. We investigated cup migration, periprosthetic bone mineral density (BMD), and patient-reported outcome measures (PROMs) until 24 months postoperative follow-up.

Results — At 24 months mean proximal cup migration was 0.11 mm (95% CI 0.00–0.23) for cemented cups and 0.09 mm (CI –0.09 to 0.28) for cementless cups. However, cementless cups generally migrated more than cemented cups at 12 and 24 months. Cemented cups had no measurable migration from 3 months’ follow-up, while cementless cups had not yet stabilized at 24 months in all rotations. Cementless cups showed statistically significantly more maximum total point motion (MTPM) at 12- and 24-month follow-up compared with cemented cups in patients with low systemic BMD (p = 0.01). Periprosthetic BMD changes did not statisticially significantly correlate to proximal migration in either cup fixation group (p > 0.05). PROMs improved similarly in both groups.

Interpretation — Cemented cups were well fixed at 3 months. The cementless cups migrated more in patients with low BMD, showed an inconsistent pattern of migration, and migrated in different directions during the first and second year without tendency to stabilization. Cemented fixation of the Avantage DM cup seems safer in elderly patients

The most common indication for revision of a conventional primary total hip arthroplasty (THA) is aseptic loosening of the components (SHAR 2016, NJR 2017, DHAR 2018).

Implant fixation method (i.e., cemented or cementless) in primary THA seems mainly based on the surgeon’s preference and national trends. The Danish Hip Replacement Registry report shows a decrease in the use of cemented cup fixation in osteoarthrosis (OA) patients above 70 years (DHAR 2018). This trend has also been described in the United Kingdom (UK) and Australian Joint Registries, while in Sweden and Norway cemented cup fixation is still the preferred fixation method in elderly patients (SHAR 2016, NAR 2017, NJR 2017).

The dual-mobility (DM) concept, with 2 articulation surfaces and increased jump distance, may decrease the dislocation rate and increase range of motion compared with standard single mobility (SM) THAs. The long-term survival and the best fixation method of the newer Avantage Reload DM cup in elderly patients is currently unknown but retrospective studies on other types of primary DM THAs suggest acceptable survival rates (Batailler et al. 2017) .

Excessive early (2-year) implant micromotion measured with radiostereometric analysis (RSA) is a strong predictor for later implant loosening and poor survival (Karrholm et al. 1997, Nieuwenhuijse et al. 2012, Pijls et al. 2012), and our primary aim was to investigate the early RSA-measured migration of cemented and cementless Avantage DM cups in elderly (> 70 years old) OA patients until 24 months’ follow-up. Secondary endpoints included systemic and periprosthetic bone mineral density (BMD) measurements, and clinical outcome scores.

## Patients and methods

### Design and patients

**Figure 1.**
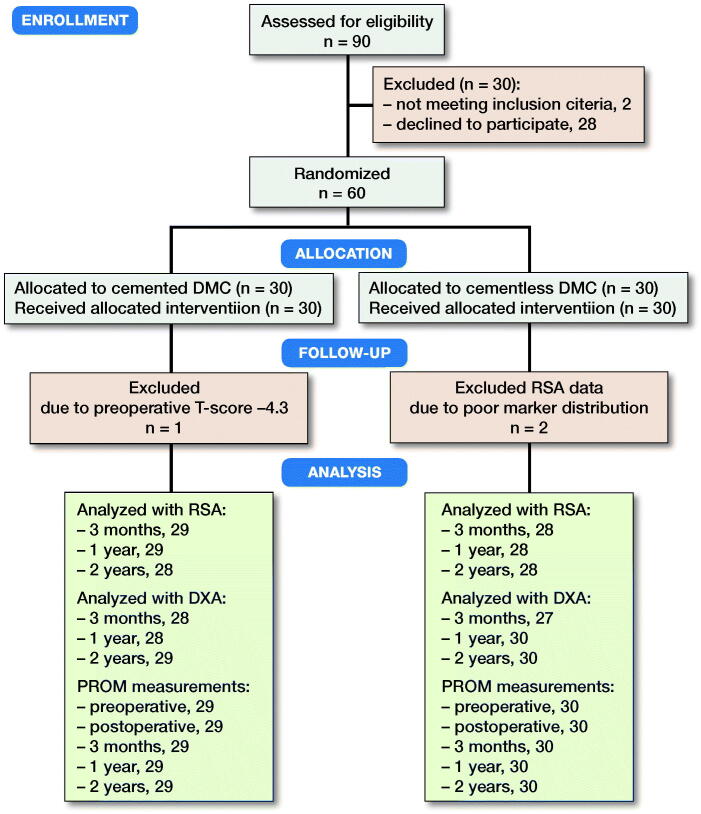
CONSORT flow diagram showing the inclusion/exclusion process until 2-year follow-up.

Between November 2014 and January 2018, we performed a Level I prospective, randomized, patient-blinded, parallel group trial at Regional Hospital West Jutland, Holstebro, Denmark. Inclusion criteria were primary osteoarthrosis, age above 70 years, informed consent, and only 1 hip operated and with adequate bone quality for insertion of a cementless acetabular component as judged by the surgeons on radiographs and intraoperatively. Exclusion criteria were vascular or neuromuscular disease in the operated leg, fracture sequelae, avascular necrosis of the femoral head, alcohol abuse, and severe osteoporosis (T-score ≤ –4.0). 60 patients (33 female) were included and block randomized (using a computerized algorithm) to either cemented (n = 30) or cementless (n = 30) cup fixation (Figure 1). There is no “acceptance limit” for “low systemic BMD/T-score.” We arbitrarily chose a T-score limit of –4.0 and thus excluded patients with severe osteoporosis.

### Prosthesis, surgery, and rehabilitation

The Avantage Reload cemented and cementless DM cup (ZimmerBiomet, Warsaw, IN, USA) has been commercially available since 2005. The external surface of the cemented Avantage Reload metal shell has a bright polish (Ra max 0.4 µm), and the inner articulate surface is highly polished. Vacuum-mixed Palacos R + G bone cement (Heraeus Medical, Wehrheim, Germany) was used for cemented fixation. The cementless Avantage Reload metal shell has a double coating with a projection vacuum plasma (VPS) titanium coating (Ra > 15 µm) and synthetic hydroxyapatite (HA) (150 ± 50 µm) to create a rough surface finish (Ra > 11 µm). Exeter highly-polished stems (Stryker Corporation, Kalamazoo, MI, USA) with vacuum mixed Palacos R + G bone cement (Heraeus Medical, Wehrheim, Germany) were used in all patients. A 28-mm chrome-cobalt femoral head was used in all cases. Vitamin E-infused highly cross-linked polyethylene liner (GUR 1050) was used in both cemented and cementless cups. All liners were vacuum-packed and gamma sterilized with a minimum of 25 kGy.

All patients were operated by 1 of 2 highly experienced orthopedic hip surgeons. The sequentially numbered sealed envelopes were hidden from investigators until directly prior to surgery to prevent bias. On the day of surgery, a sealed randomization envelope was opened to allocate the patient to either cemented or cementless cup fixation. Prophylactic cefuroxime 1.5 g was administrated intravenously before surgery and twice postoperatively with an 8-hour interval. After bone preparation, 6–8 tantalum beads (1 mm) were inserted into the periacetabular bone during surgery. All patients were operated by a posterolateral approach and received the same rehabilitation program, allowing full weight-bearing immediately after surgery.

### Radiostereometric analysis

Stereoradiographs were obtained within the first postoperative 2 days (mean 1.1, range 1–14) and at 3, 12, and 24 months after surgery. All examinations were performed with the patient in a supine position with a uniplanar calibration box (Carbon Box 19, RSAcore, Leiden, The Netherlands) located underneath the examination table. The anatomical axis of the leg was parallel to the y-axis of the calibration box. Cup migration was evaluated on all 3 follow-up stereoradiographs with the postoperative stereoradiograph as the baseline reference.

**Figure 2.**
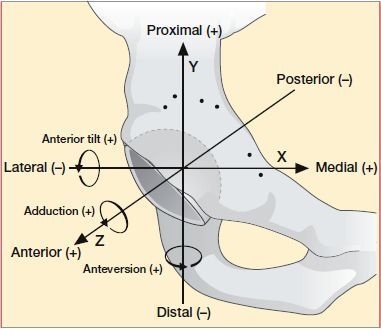
Illustration of directions, translation, and rotations for Avantage DM cup.

The radiostereometric analysis was performed with Model-Based RSA version 4.10 software (RSAcore, Leiden, The Netherlands) using computer-aided design (CAD) implant models provided by the manufacturer (ZimmerBiomet, Warsaw, IN, USA). We measured cup migration (center of 3D model points) in the coordinate system of the calibration box as described in the Guidelines for RSA of Implants (Valstar et al. 2005) (Figure 2). Total translation (TT) and total rotation (TR) were both calculated using Pythagoras’ theorem (sqrt (x^2^ + y^2^ + z^2^). The mean condition number (CN) of the bone marker model was 83 (SD 47) and the rigid body error (ME) was mean 0.24 (SD 0.06). A minimum of 3 bone markers was accepted and the cut-off points for CN and ME were maintained at 150 and 0.35, respectively (Valstar et al. 2005).

All patients were subject to double examinations at the 3-month RSA examination according to guidelines (Table 1, see Supplementary data) (Valstar et al. 2005).

**Table 1.** RSA measurement error based on double-examination stereoradiographs

	Translation (mm)	Rotation (°)							
	X	Y	Z	TT^a^	X	Y	Z	TR^b^	MTPM^c^
Mean dif.^d^	0.02	–0.01	–0.01	0.00	–0.23	–0.05	0.09	0.07	0.01
SD dif.^e^	0.20	0.09	0.16	0.17	0.91	0.92	0.64	0.90	0.57
CR (1.96 × SD dif.)^f^	0.39	0.18	0.31	0.33	1.78	1.80	1.25	1.76	1.12

There was no statistically significant difference between cemented and cementless fixation.

a TT: total translation was calculated using 3D Pythagorean theorem (TT = sqrt (xt2 + yt2+ zt2).

b TR: total rotation was calculated using 3D Pythagorean theorem (TR = sqrt (xr2 + yr2 + zr2).

c MTPM: maximum total point motion is an absolute migration parameter (migration vector).

d Mean dif.: systematic error between two RSA double measurements (should optimally be 0).

e SD dif.: standard deviation of the difference between the two examinations (SD dif.) reflects the precision of the applied RSA method.

f CR: the coefficient of repeatability (1.96 × SD dif.) reflects the lower limit within which it is possible to detect prosthetic migration on an individual basis.

The position of the fitted implant CAD model on the postoperative stereoradiograph pose-estimation served as inclination and anteversion estimates and were read from the Model-Based RSA software (RSAcore, Leiden, The Netherlands).

### Dual-energy X-ray absorptiometry (DXA) scans

**Figure 3.**
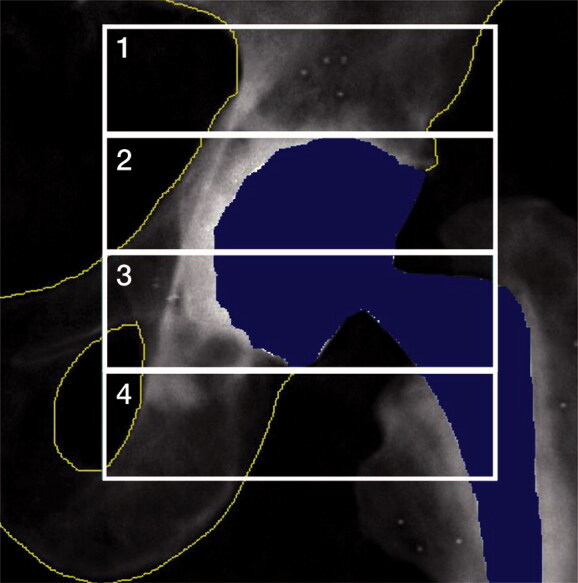
Wilkinson regions of interest (ROI) 1–4. Only the bone areas within yellow lines are included in the analysis.

Preoperatively all patients underwent spine and dual hip DXA scan to determine systemic BMD and T-score. The mean time from preoperative DXA scan to surgery was 20 days (14–71). Postoperatively (within 4 days after surgery) and at 3, 12, and 24 months after surgery, quantitative measurements of the periprosthetic BMD (g/cm^2^) was acquired with DXA scans using a GE Lunar iDXA scanner (General Electric, Chicago, IL, USA), and analyses were performed using enCORE version 16 software (https://www.encore.com/). Patients were placed in supine position with the body parallel to the examination table and the feet fixed to a device that kept the halluces pointing straight up. Analysis was performed according to Wilkinson 4 regions of interest (ROI) and precision ranged from 3% to 13% in cemented cup fixation and 3% to 6% in cementless cup fixation (Table 2, see Supplementary data) (Wilkinson et al. 2001). A template was applied to the baseline scan, and the ROIs were subsequently copied to align with the bone-border on follow-up scans. ROI 2 and 3 were adjusted in height on the baseline scan depending on the cup size (each ROI was 1 half cup height) and ROIs 1 and 4 had fixed sizes (Figure 3).

**Table 2.** DXA (g/cm^2^) measurement error based on 3-month double-examination DXA scans for cemented and cementless cup fixation

	Cemented	Cementless						
	ROI1	ROI2	ROI3	ROI4	ROI1	ROI2	ROI3	ROI4
Mean dif.	–0.02	–0.01	–0.05	–0.01	0.02	–0.01	0.00	0.01
SD dif.	0.07	0.31^a^	0.13^a^	0.07	0.07	0.11^a^	0.07^a^	0.05
CV %^b^	3.02	12.50	8.20	5.40	3.20	6.26	5.83	4.14

a Denotes significant difference between cemented and cementless cups using the F-test.

b CV% = 100 Ч [(δ/√2)/µ] for each ROI for cemented and cementless cup fixation.δ represents the SD of the difference between the paired BMD measurements, and µ is the overall mean of all BMD measurements for each ROI.

### Clinical outcome measures and complications

Clinical outcome measures were assessed by Harris Hip Score (HHS), Oxford Hip Score (OHS) (Paulsen et al. 2012), patient-reported quality of life (EQ-5D) (Brooks 1996), and visual analog scale (VAS) for hip pain preoperatively and at 3, 12, and 24 months after surgery.

### Statistics and sample size

The primary endpoint was proximal cup migration at the 24-month follow-up (Pijls et al. 2012). Linear mixed-effect models were used to determine whether cup fixation and BMD had a significant effect on cup migration at 3-, 12-, and 24-month follow-up. This was used as it takes the correlation of measurements on the same patient into account and includes all patients/missing values effectively. The analysis was modelled as a function of fixation with the interaction of time and fixation as fixed effects. Bonferroni correction was applied to migration data (primary end-point), when linear mixed models showed significant p-values. Model estimates are reported as means with 95% confidence intervals (CI). The secondary endpoints were measurements of periprosthetic BMD, clinical outcomes of HHS, OHS, and EQ-5D, and VAS (rest and activity) for pain. Subgroup analyses (mixed model) were performed between cup fixation (cemented/cementless) and cup migration when stratified to normal (T-score ≥ –1.0) or low (T-score < –1.0) preoperative BMD. Student’s t-test was used for normally distributed data. When data were not normally distributed according to a Shapiro–Wilks test, a non-parametric (Mann–Whitney) test was used. Data were analyzed as of the date of the last data collection (January 2018).

Proximal cup acceptance migration thresholds at 24 months’ follow-up was assessed according to Pijls et al. (2012).

There are no previous migration data for the Avantage DM cup that could be used for sample size calculation. The pilot study included both cemented and cementless cups in patients (n = 5) older than 70 years. A pre-study sample size calculation using 2-sample mean test for a minimal relevant proximal cup migration difference of 0.2 mm (Pijls et al. 2012) with a mean cup migration of 0.2 mm (SD 0.27) (pilot study), power 80%, alpha 0.05, estimated 29 patients in each group. 30 patients per group were included to account for dropout. Statistical significance was set at 0.05. Stata version 13.1 (StataCorp, College Station, TX, USA) was used for statistical analysis.

### Ethics, funding, and potential conflicts of interest

The study was conducted in accordance with the Helsinki Declaration. Patients gave informed consent before entering the study. The study was approved by the Central Danish Regional Committees on Biomechanical Research Ethics (Journal no. 1-10-72-209-14) and the study was registered with ClinicalTrials.gov (NCT02404727). ZimmerBiomet Inc. and The Danish Rheumatism Association supported the study financially but had no influence on the manuscript or publication. The authors have no conflicts of interest.

## Results

The baseline demographics of all patients are presented in Table 3.

**Table 3.** Descriptive baseline characteristics of the patients, implants, and surgery. Values are mean (range) unless otherwise specified

	Cemented	Cementless
	(n = 29)	(n = 30)
Sex, male/female, n	14/15	13/17
Age at operation	75 (70–82)	75 (70–83)
Implant side, right/left, n	16/13	15/15
Cup size (mm)	48.7 (44–54)	52.8 (48–58)
Cup inclination (°)	49.2 (36.2–61.0)	43.5 (28.9–59.7)
Cup anteversion (°)	11.5 (1.2–26.2)	11.7 (0.7–26.3)
Preoperative T-score	–1.01 (–2.9–1.8)	–1.12 (–3.1–2.3)
BMI	28 (23–39)	29 (22–38)
ASA class	2.0 (1–3)	1.8 (1–3)

### Radiostereometric analysis

**Figure 4.**
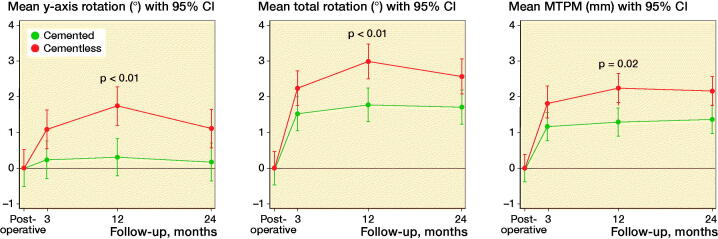
Significant migration in cementless cups compared with cemented in y-axis, TR, and MTPM.

Translations and rotations, including TT, TR, and MTPM (mean and CI), are presented in Table 4, and statistically significant migrations are presented in Figure 4. Cemented cups showed no statistically significant translation (p > 0.3) or rotation (p > 0.2) during the 24-month follow-up time. Cementless cups had no statistically significant translations (p > 0.20) during 24-month follow-up, but showed rotation about all orthogonal axes and in TR during the 24-month follow-up (Table 5). Cemented cups migrated 0.54 (CI 0.44–0.65) and cementless cups migrated 1.08 (CI 0.71–1.46) MTPM between 12 months and 24 months with a mean difference of –0.54 (CI –0.94 to –0.14, (p = 0.01).

**Table 4.** Translations along and rotations about the x-, y-, and z-axis for cemented and cementless cups presented as mean (95% CI)

Cemented		Cementless
Axis	(n = 28)	(n = 28)
Translations (mm)		
x-axis		
3 months	–0.01 (–0.17 to 0.14)	0.08 (–0.19 to 0.36)
12 months	–0.03 (–0.21 to 0.15)	0.16 (–0.20 to 0.51)
24 months	–0.01 (–0.22 to 0.20)	0.23 (–0.20 to 0.66)
y-axis		
3 months	0.08 (0.00 to 0.16)	0.15 (0.02 to 0.27)
12 months	0.09 (0.01 to 0.18)	0.12 (–0.02 to 0.26)
24 months	0.11 (0.00 to 0.23)	0.09 (–0.09 to 0.28)
z-axis		
3 months	0.16 (0.00 to 0.32)	0.31 (0.00 to 0.62)
12 months	0.15 (–0.01 to 0.31)	0.36 (0.03 to 0.69)
24 months	0.23 (0.02 to 0.44)	0.39 (0.03 to 0.75)
Total translation		
3 months	0.49 (0.34 to 0.64)	0.79 (0.49 to 1.10)
12 months	0.56 (0.37 to 0.76)	0.88 (0.51 to 1.25)
24 months	0.65 (0.44 to 0.87)	0.98 (0.54 to 1.42)
Rotations (°)		
x-axis		
3 months	0.34 (0.01 to 0.66)	0.01 (–0.48 to 0.51)
12 months	0.52 (0.15 to 0.89)	0.64 (–0.01 to 1.30)
24 months	0.29 (–0.05 to 0.63)	0.04 (–0.63 to 0.70)
y-axis **^a^**		
3 months	0.23 (0.26 to 0.72)	1.08 (0.34 to 1.82)
12 months	0.30 (–0.25 to 0.85)	1.74 (0.91 to 2.57) **^b^**
24 months	0.18 (–0.37 to 0.73)	1.10 (0.42 to 1.78)
z-axis		
3 months	–0.35 (–0.60 to 0.03)	–0.07 (–0.60 to 0.46)
12 months	–0.40 (–0.75 to –0.05)	–0.33 (–0.92 to 0.26)
24 months	–0.35 (–0.76 to 0.05)	–0.01 (–0.69 to 0.68)
Total rotation ^a^		
3 months	1.52 (1.12 to 1.90)	2.23 (1.55 to 2.92)
12 months	1.80 (1.40 to 2.24)	3.00 (2.20 to 3.80) **^b^**
24 months	1.72 (1.30 to 2.13)	2.57 (1.83 to 3.30)
MTPM (mm) ^a^		
3 months	1.14 (0.86 to 1.42)	1.81 (1.26 to 2.36)
12 months	1.30 (1.00 to 1.60)	2.24 (1.64 to 2.85) **^b^**
24 months	1.36 (1.00 to 1.73)	2.16 (1.44 to 2.87)

MTPM: maximum total point motion.

**^a^**Denotes Bonferroni adjusted p-values for multiple testing.

**^b^**Statistically significant difference between cemented and cementless cup fixation.

**Table 5.** Cup rotations and MTPM between follow-ups within each cup fixation group presented as mean difference (95% CI)

Rotations (°)	Cemented	Cementless
x-axis		
3–12 months	–0.18 (–0.49 to 0.13)	–0.63 (–0.95 to 0.31)^a^
12–24 months	0.23 (–0.08 to 0.55)	0.61 (0.28 to 0.93)^a^
y-axis		
3–12 months	–0.07 (–0.44 to 0.29)	–0.66 (–1.03 to 0.28)^a^
12–24 months	0.14 (–0.22 to 0.51)	0.64 (0.26 to 1.01)^a^
z-axis		
3–12 months	0.09 (–0.15 to 0.32)	0.26 (0.01 to 0.50)
12–24 months	–0.08 (–0.32 to 0.15)	–0.33 (–0.60 to –0.08)^a^
Total translation		
3–12 months	–0.25 (–0.62 to 0.12)	–0.75 (–1.13 to 0.36)^a^
12–24 months	0.07 (–0.31 to 0.44)	0.42 (0.04 to 0.80)^a^

a Statistically significant within-group difference from one follow-up to next follow-up.

**Figure 5.**
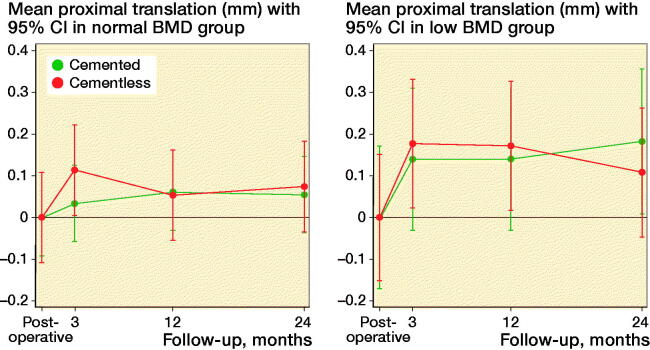
Proximal translation in normal and low BMD when stratified according to fixation method.

By 24 months, 21/28 of the cemented cups showed proximal cup migration < 0.2 mm, 7/28 were between 0.2 and 1.0 mm, and no cemented cups had proximal cup migration > 1.0 mm. By 24 months, 18/28 of the cementless cups showed proximal cup migration < 0.2 mm, 9/28 were between 0.2 and 1.0 mm, and 1/28 cementless cup showed > 1.0 mm proximal cup migration. When stratifying patients into 2 subgroups based on preoperative systemic BMD (normal and low BMD), we found no within-subgroup difference in proximal cup migration between cemented and cementless cup fixation at any follow-up (p > 0.3; Figure 5, see Supplementary data). The mean 24-month proximal cup migration in the normal BMD group was 0.05 mm (CI –0.08– 0.18) for cemented cups and 0.07 mm (CI –0.17–0.32) for cementless cups (Figure 5, see Supplementary data). Mean 24-month proximal cup migration in the low BMD group was 0.18 mm (CI 0.05–0.31) for cemented cups and 0.11 mm (CI –0.07–0.29) for cementless cups (Figure 5, see Supplementary data).

The postoperative inclination angle was higher in cemented cups compared with cementless cups (p = 0.01) (Table 3). The postoperative anteversion angle did not differ between the two fixation methods (p = 0.9) (Table 3).

**Figure 6.**
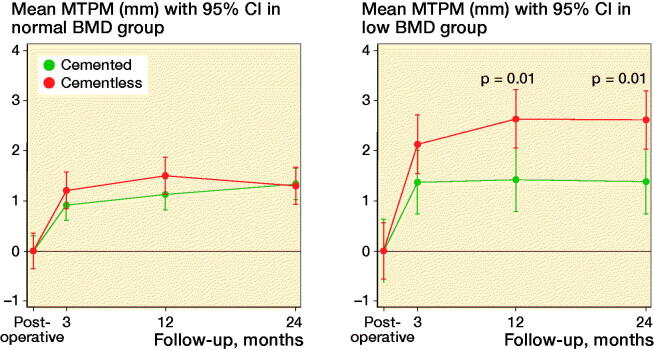
MTPM migration in normal and low BMD groups based on cup fixation.

Further analyses on BMD groups showed that MTPM was statistically significantly higher at 12- and 24-month follow-up in cementless cups compared with cemented cups in the low BMD group (p = 0.01, Figure 6), which could be explained by a higher cup migration in x-translation (p = 0.04 at 24 months), y- rotation (p < 0.001, p = 0.03, at 12 and 24 months respectively), and z-rotation (p = 0.04 at 24 months). Likewise, TT and TR was higher for cementless cups compared with cemented cups in the low BMD groups at 12 and 24 months (all p < 0.03).

### Percentage change in periprosthetic BMD

Percentage BMD changes are presented in Table 6. We did not find a statistically significant correlation between percentage BMD change and proximal cup migration in cemented or cementless cups during follow-up (p > 0.06). However, this does not imply that there is no true correlation between percentage BMD change and proximal cup migration. The strongest correlation between proximal cup migration and percentage BMD was found at 24 months in the cementless group in ROI 4 (CI –0.68–0.00, p = 0.06).

**Table 6.** BMD change in the 4 ROIs around the acetabular component presented as mean (95% CI) percentage change from the baseline values at 3, 12, and 24 months

ROI Follow-up	Cemented	Cementless	
ROI 1	Postoperative	ref	ref
	3 months	0 (–2 to 2)	–1 (–4 to 1)
	12 months	1 (–1 to 3)	–2 (–4 to 0)
	24 months	3 (1 to 5)	–2 (–4 to 0) **^a^**
ROI 2	Postoperative	ref	ref
	3 months	–7 (–13 to –1)	–4 (–11 to 2)
	12 months	–1 (–6 to 6)	–12 (–19 to –6) **^a^**
	24 months	–7 (–13 to –1)	–11 (–17 to –4)
ROI 3	Postoperative	ref	ref
	3 months	–5 (–10 to 0)	4 (–1 to 9) **^a^**
	12 months	–2 (–7 to 3)	4 (–1 to 8)
	24 months	–1 (–6 to 4)	4 (–1 to 9)
ROI 4	Postoperative	ref	ref
	3 months	–5 (–8 to –1)	–1 (–4 to 3)
	12 months	–7 (–10 to –3)	–2 (–6 to 2)
	24 months	–9 (–13 to –6)	–1 (–4 to 3) **^a^**

ROI: Regions of interest according to Wilkinson et al. (2001).

a Statistically significant difference between cemented and cementless fixation.

### Clinical outcome measures and complications

Clinical results (HHS, OHS, EQ-5D, VAS) were similar between the 2 fixation groups during 24-month follow-up (Table 7, see Supplementary data). The 2 groups showed similar clinical improvement from preoperative to 24-month follow-up. The biggest difference between fixation groups was found in VAS at activity, mean diff = 1.0 (CI 0.02–2.1, p > 0.07).

**Table 7.** Mean (SD) scores for the HHS, OHS, EQ-5D, and VAS for pain

	Cemented	Cementless		
Outcomes	(n = 29)	(n = 30)	p-value	
HHS				
	Preoperative	56 (12)	56 (16)	0.6
	3 months	80 (13)	81 (14)	0.6
	12 months	92 (6.5)	89 (10)	0.3
	24 months	92 (8.7)	90 (11)	0.7
OHS				
	Preoperative	25 (6.5)	25 (6.2)	0.8
	3 months	37 (8.0)	39 (5.6)	0.8
	12 months	45 (3.9)	43 (4.9)	0.1
	24 months	45 (4.3)	43 (5.5)	0.3
EQ-5D				
	Preoperative	0.63 (0.15)	0.66 (0.10)	0.9
	3 months	0.88 (0.13)	0.90 (0.10)	0.6
	12 months	0.93 (0.10)	0.92 (0.11)	0.8
	24 months	0.94 (0.10)	0.92 (0.10)	0.4
VAS for hip pain (rest)				
	Preoperative	3.2 (2.7)	2.9 (2.0)	0.7
	3 months	0.9 (1.3)	0.7 (0.8)	0.6
	12 months	0.03 (0.2)	0.2 (1.1)	0.5
	24 months	0.1 (0.6)	0.2 (0.8)	0.6
VAS for hip pain (activity)				
	Preoperative	6.8 (1.9)	5.5 (2.1)	0.02
	3 months	1.0 (0.9)	0.9 (0.8)	0.7
	12 months	0.2 (0.5)	0.5 (1.4)	0.5
	24 months	0.4 (1.0)	0.1 (0.3)	0.4

HHS: Harris Hip Score. OHS: Oxford Hip Score.

EQ-5D: EuroQol—five-dimensional. VAS: Visual Analogue Scale.

1 patient (cementless cup) underwent revision surgery (liner and femoral head exchange) 3 months after the index surgery due to an intraprosthetic dislocation. 2 weeks after revision, the patient underwent a 1-stage debridement, washout, femoral head and liner exchange, and antimicrobial treatment for 6 weeks due to deep infection (*Staphylococcus aureus*). Here­after, the patient had a well-functioning hip and continued regular RSA follow-up. There were no large articulation dislocations in either fixation group during the follow-up period.

## Discussion

To our knowledge, this is the first RSA study of the DM articulation concept in elderly OA patients comparing cemented and cementless cup fixation in a randomized study. The key findings were similar proximal cup migration between fixation groups, but cementless cups migrated more on absolute measures, migrated more in patients with low BMD, and had not stabilized at 24 months, whereas cemented cups were stable from 3 months.

### Radiostereometric analysis

The association between early high proximal cup migration and the elevated risk of aseptic cup loosening and later revision have previously been described (Nieuwenhuijse et al. 2012, Pijls et al. 2012). In relation to Pijls’ (2012) thresholds for proximal cup migration, we identified 7 cemented cups (range 0.23–0.71 mm) and 9 cementless cups (range 0.2–0.75 mm) “at risk” of later revision in our study, but we observed no cemented cups and 1 cementless cup (1.16 mm) with “unacceptable” proximal migration (Pijls et al. 2012). In relation to Nieuwenhuijse’s definition we observed no cups exceeding 1.76 mm proximal migration and 1 cementless cup (6.39°) with abduction (z-axis) above 2.53° (Nieuwenhuijse et al. 2012).

Cementless cups are inserted by under-reamed technique, and the initial rim-fit may be lost over time resulting in a final bottoming in the acetabulum (Rohrl et al. 2004). However, we saw 1 cementless cup with large proximal migration, and in general no measurable translation over time in the cementless group. Cementless cups did, however, have more rotation overall, over time, and in opposite directions before and after 12 months, as compared with cemented cups. A clinical significance level in relation to rotation measures has not been established. The difference in cup y-rotation was evident at 3 months and was probably an effect of final osseointegration.

Cemented cups were inserted with significantly higher inclination angle compared with cementless cups, which may be explained by surgeons’ preference for free-hand insertion of cemented Avantage DM cups due to affection of the cup–cement interface before cement curing with disconnection of the cup-guide. However, our findings suggest that bone fixation of cemented cups is less sensitive to increased cup angulation compared with cementless cups. This is also in line with a study on all-poly cemented and cementless cups (Kadar et al. 2012).

RSA evaluations of elderly patients with OA treated with a primary THA are scarce. Direct comparisons with previous RSA reports are difficult due to alternative ways of presenting data, methods of fixation, marked differences in patient demographics, implant design, surgical approach, and follow-up time. Based on 24-month proximal migration as an indicator for primary stability our findings for cemented and cementless fixation methods are comparable, and in many cases lower than reported in other studies on cemented and cementless cup fixation in primary THA (Lazarinis et al. 2014, Salemyr et al. 2015, Finnila et al. 2016).

### Radiostereometric analysis and preoperative BMD status

Cementless ceramic-on-ceramic THA has shown statistically significantly and clinically relevant higher proximal migration and migration until 12-month follow-up in women with low BMD (lower T-score limit of –3.5) compared with women with normal BMD at 24-month follow-up (Finnila et al. 2016). These findings are inconsistent with the present study where we observed similar proximal migration in the normal BMD and low BMD groups and no migration within fixation groups of the BMD subgroups during follow-ups. The mean 24-month proximal migration in our cementless group with low BMD of 0.11 mm (CI –0.07 to 0.29) was lower than reported in the study by Finnilä et al. (2016) of 0.29 mm (CI 0.20–0.39), suggesting early initial proximal cup stability with Avantage DM cups, even in the low BMD group, and in both cemented and cementless cup fixation. However, in the low BMD group, cementless cups showed statistically significantly more migration in MTPM, x-axis translation, y-axis rotation, TT and TR compared with cemented cups, suggesting that cementless cup fixation is not preferable in patients with preoperative low BMD. Only 1 study reports proximal cup migration in cemented cups with stratification to normal and low BMD but unclear definition of osteoporosis makes direct comparison troublesome (Digas et al. 2004).

### Periprosthetic BMD measurements

Differences in BMD change in cemented and cementless fixation may be a result of different load transfer mechanisms leading to different bone remodeling profile (Digas et al. 2006). In cementless cups, forces are transmitted sideways to the periphery, rather than proximally, which leads to reduced load transfer in the most cranial/proximal area (Digas et al. 2006, Lazarinis et al. 2014) with local bone resorption caused by stress-shielding. This might explain the greater bone loss observed in ROI 1 and 2 of cementless cups compared with cemented cups in our study. Conversely, the increased BMD in ROI 3 and lesser BMD reduction in ROI 4 in cementless cups compared with cemented could be due to the increased traction forces in cementless cups acting as a stimulus for preservation of bone or even increase in BMD (Salemyr et al. 2015). The percentage BMD changes in the 2 cup fixation methods did not correlate to proximal cup migration.

### Clinical outcome measures

There was no statistically significant difference in postoperative clinical evaluations (quality of life measured by EQ-5D, or hip status measured by HHS and OHS) between cemented and cementless cup groups. The 2-year clinical evaluations of cemented and cementless fixation translates to either very good or excellent end-results (Nilsdotter and Bremander 2011).

### Cup fixation method in the elderly

There is no clear consensus on the choice of the cemented or cementless cup fixation method in elderly patients, and registry reports from the UK, Australia, Sweden, Norway, and Denmark reveal no clear overall tendency regarding cup fixation methods in the elderly (SHAR 2016, NAR 2017, NJR 2017, DHAR 2018). While many registries report a tendency towards more cups being inserted with cementless fixation, their superiority is not supported in the literature (Troelsen et al. 2013, Makela et al. 2014).

### Limitations and strengths

The strength of this study is the patient-blinded randomized controlled study design and a large group available for migration analysis. RSA is a validated surrogate measure of later implant loosening, but other complications, i.e., wear-induced osteolysis or fractures in the cement mantle, may not be detected with early RSA (Nieuwenhuijse et al. 2012). Linear mixed-model analysis enabled us to use all the available data. A high number of radiographs were available for analysis, and the number of patients with available 24-month RSA measurements was equal in both fixation groups. Only 2 patients in the cementless group were excluded due to poor marker distribution. In the cemented group 1 patient was excluded due to a mistake in identification of severe preoperative osteoporosis (preoperative T-score of –4.3) and the 24-month follow-up RSA radiographs of 1 patient were accidentally lost. BMD is typically higher around cemented cups due to opacity of the cement and therefore net BMD measurements are not direct comparable between cementless and cemented cups, and for this reason we compared percentage BMD changes (Jayasuriya and Wilkinson 2014). The high inclusion rate improves the external validity of our study, but results can be generalized only to patients older than age 70 years with a T-score above –4.

## Conclusion

At minimum 2 years’ follow-up cemented and cementless DM cups had similar proximal mean migration below the recommended acceptance threshold, yet more cups in the cementless group migrated above the acceptance levels, which is of clinical importance. The cementless cups migrated more in patients with low BMD, showed an inconsistent pattern of migration, and migrated in different directions during the first and second year without tendency to stabilization. On the other hand, cemented cups were well fixed at 3 months. Cemented fixation of the Avantage DM cup seems safer in elderly patients; however, long-term studies are warranted.

## Supplementary Material

Supplemental Material

## Supplementary data

Tables 1, 2, 7, and Figure 5 are available as supplementary data in the online version of this article, http://dx.doi.org/ 10.1080/17453674.2020.1720978

ST-J, TBH, and MS designed the study and validated the methods. ST-J performed all the follow-up examinations. SBM and ST-J performed the statistical analyses. ST-J wrote the initial manuscript, and all authors revised it.

Note: All product names are for identification purposes only, and may be trademarks of their respective owners.

*Acta* thanks Søren Kold and Johan Kärrholm for help with peer review of this study.
