# Supplementary material for: Inferior stabilization of cementless compared with cemented dual-mobility cups in elderly osteoarthrosis patients: a randomized controlled radiostereometry study on 60 patients with 2 years’ follow-up
Source: Acta Orthop. 2020 Feb 6;91(3):246–53. doi: 10.1080/17453674.2020.1720978 (PMC8023886; doi:10.1080/17453674.2020.1720978)
Supplement: Supplemental Material [file IORT_A_1720978_SM4691.pdf]

Supplementary data

Table 1. RSA measurement error based on double-examination stereoradiographs

	Translation (mm)				Rotation (°)				
	X	Y	Z	TT [a]	X	Y	Z	TR [b]	MTPM [c]
Mean dif. [d]	0.02	–0.01	–0.01	0.00	–0.23	–0.05	0.09	0.07	0.01
SD dif. [e]	0.20	0.09	0.16	0.17	0.91	0.92	0.64	0.90	0.57
CR (1.96 × SD dif.) [f]	0.39	0.18	0.31	0.33	1.78	1.80	1.25	1.76	1.12

There was no statistically significant difference between cemented and cementless fixation.
[a] TT: total translation was calculated using 3D Pythagorean theorem (TT = sqrt (xt2 + yt2+ zt2).
[b] TR: total rotation was calculated using 3D Pythagorean theorem (TR = sqrt (xr2 + yr2 + zr2).
[c] MTPM: maximum total point motion is an absolute migration parameter (migration vector).
[d] Mean dif.: systematic error between two RSA double measurements (should optimally be 0).
[e] SD dif.: standard deviation of the difference between the two examinations (SD dif.) reflects the precision of the applied RSA method.
[f] CR: the coefficient of repeatability (1.96 × SD dif.) reflects the lower limit within which it is possible to detect prosthetic migration on an individual basis.

Table 2. DXA (g/cm^2) measurement error based on 3-month double-examination DXA scans for cemented and cementless cup fixation

	Cemented				Cementless			
	ROI1	ROI2	ROI3	ROI4	ROI1	ROI2	ROI3	ROI4
Mean dif.	–0.02	–0.01	–0.05	–0.01	0.02	–0.01	0.00	0.01
SD dif.	0.07	0.31 [a]	0.13 [a]	0.07	0.07	0.11 [a]	0.07[a]	0.05
CV % [b]	3.02	12.50	8.20	5.40	3.20	6.26	5.83	4.14

[a] Denotes significant difference between cemented and cementless cups using the F-test.
[b] CV% = 100 × [(δ/√2)/μ] for each ROI for cemented and cementless cup fixation.
δ represents the SD of the difference between the paired BMD measurements, and μ is the overall mean of all BMD measurements for each ROI.

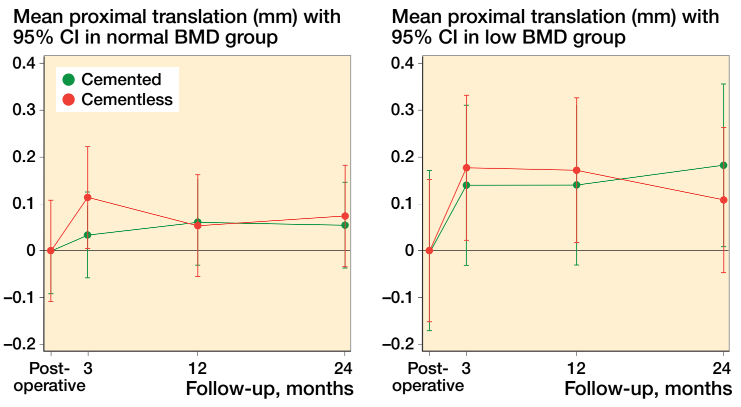

Figure 5. Proximal translation in normal and low BMD when stratified according to fixation method.

Table 7. Mean (SD) scores for the HHS, OHS, EQ-5D, and VAS for pain

Outcomes	Cemented (n = 29)	Cementless (n = 30)	p-value
HHS			
Preoperative	56 (12)	56 (16)	0.6
3 months	80 (13)	81 (14)	0.6
12 months	92 (6.5)	89 (10)	0.3
24 months	92 (8.7)	90 (11)	0.7
OHS			
Preoperative	25 (6.5)	25 (6.2)	0.8
3 months	37 (8.0)	39 (5.6)	0.8
12 months	45 (3.9)	43 (4.9)	0.1
24 months	45 (4.3)	43 (5.5)	0.3
EQ-5D			
Preoperative	0.63 (0.15)	0.66 (0.10)	0.9
3 months	0.88 (0.13)	0.90 (0.10)	0.6
12 months	0.93 (0.10)	0.92 (0.11)	0.8
24 months	0.94 (0.10)	0.92 (0.10)	0.4
VAS for hip pain (rest)			
Preoperative	3.2 (2.7)	2.9 (2.0)	0.7
3 months	0.9 (1.3)	0.7 (0.8)	0.6
12 months	0.03 (0.2)	0.2 (1.1)	0.5
24 months	0.1 (0.6)	0.2 (0.8)	0.6
VAS for hip pain (activity)			
Preoperative	6.8 (1.9)	5.5 (2.1)	0.02
3 months	1.0 (0.9)	0.9 (0.8)	0.7
12 months	0.2 (0.5)	0.5 (1.4)	0.5
24 months	0.4 (1.0)	0.1 (0.3)	0.4

HHS: Harris Hip Score. OHS: Oxford Hip Score.
EQ-5D: EuroQol—five-dimensional. VAS: Visual Analogue Scale.
